# A Table of Food Values

**Published:** 1894-03

**Authors:** W. O. Atwater


### A Table of Food Values.

| Nourishment. | Energy. |
|---|---|
| 1. Cornmeal. | 1. Cornmeal. |
| 2. Wheat flour. | 2. Wheat flour. |
| 3. Oatmeal. | 3. Oatmeal. |
| 4. Salt cod. | 4. Sugar. |
| 5. Beef (neck). | 5. Potatoes. |
| 6. Cheese. | 6. Salt pork. |
| 7. Potatoes. | 7. Wheatbread. |
| 8. Wheatbread. | 8. Cheese. |
| 9. Chicken. | 9. Butter. |
| 10. Milk. | 10. Beef (neck). |
| 11. Eggs. | 11. Milk. |
| 12. Surloin. | 12. Surloin. |
| 13. Mutton. | 13. Salt cod. |
| 14. Salmon. | 14. Mutton. |
| 15. Oysters. | 15. Eggs. |
| 16. Salt pork. | 16. Chicken. |
| | 17. Salmon. |
| | 18. Oysters. |

—W. O. Atwater, in *The Forum.*

